# Microbiota, parasitic infections and their relationship with nutritional status and neurocognitive functioning in children from Ecuador—Proyecto Guagua: research protocol for a cross-sectional study

**DOI:** 10.3389/fpubh.2025.1505780

**Published:** 2025-01-29

**Authors:** Sandra Victoria Abril-Ulloa, Tannia Valeria Carpio-Arias, Renata Alejandra Alvarado-Barba, Cristina Gabriela Ríos-Romero, Pamela Vinueza-Veloz, Sueny Paloma Lima-dos-Santos, Igor Eduardo Astudillo-Skliarova, Ruth Irene Arias-Gutiérrez, Manuel Pérez-Quintana, Henk-Jan Boele, María Fernanda Vinueza-Veloz

**Affiliations:** ^1^Carrera de Nutrición y Dietética, Facultad de Ciencias Médicas, Universidad de Cuenca, Cuenca, Ecuador; ^2^Grupo de Investigación en Alimentación y Nutrición Humana (GIANH), Facultad de Salud Pública, Escuela Superior Politécnica de Chimborazo, Riobamba, Ecuador; ^3^Grupo de Investigación en Ciencias Veterinarias (GICV), Carrera de Medicina Veterinaria, Escuela Superior Politécnica de Chimborazo, Riobamba, Ecuador; ^4^Graduate College, College of Health Sciences and Professions, Ohio University, Athens, OH, United States; ^5^Center for Nutrition and Health Impact, Omaha, NE, United States; ^6^Carrera de Medicina, Facultad de Salud Pública, Escuela Superior Politécnica de Chimborazo, Riobamba, Ecuador; ^7^Carrera de Nutrición y Dietética, Facultad de Salud Pública, Escuela Superior Politécnica de Chimborazo, Riobamba, Ecuador; ^8^Facultad de Ciencias de la Vida, Universidad Estatal Amazónica, Puyo, Ecuador; ^9^Department of Neuroscience, Erasmus MC, Rotterdam, Netherlands; ^10^Neuroscience Institute, Princeton University, Princeton, NJ, United States; ^11^BlinkLab Ltd., Sydney, NSW, Australia; ^12^Department of Community Medicine and Global Health, Institute of Health and Society, University of Oslo, Oslo, Norway

**Keywords:** malnutrition, Ecuador, children, neurocognitive abilities, parasitic infection, motor learning, social skills, microbiota

## Abstract

**Introduction:**

This protocol outlines the Proyecto Guagua, which aims to explore the relationship between the characteristics of the gut microbiota, parasitic infections, nutritional status, and neurocognitive functioning in school-age children in Ecuador.

**Methods and analysis:**

Proyecto Guagua is a cross-sectional observational study funded by the Escuela Superior Politécnica de Chimborazo. It is being carried out in several counties across different geographical regions in Ecuador, including Galápagos. The study targets children regularly attending school, aged 6–12. We aim to recruit 450 children, with data already collected from nearly 300 participants (67%). Enrolled children undergo comprehensive evaluations assessing nutritional status, body composition, motor learning, social skills, cognitive ability, sleep habits, and physical activity. Caretakers and school teachers are interviewed regarding hygiene, eating habits, and food handling. Stool samples are collected to analyze the gut microbiota and determine the presence of parasites. In the analysis phase, we aim to describe differences in microbiota population structure and diversity among undernourished and obese/overweight children, and children with parasitosis compared to their peers. We also plan to test the hypothesis that an altered microbiota mediates the influence of malnutrition on neurocognitive functioning and parasitosis.

**Ethics and dissemination:**

Proyecto Guagua received ethical approval from the Ethics Committee of Universidad de Cuenca in July 2022. Following the pilot phase, an addendum and minor changes to the study design were approved in October 2022. Written consent was obtained from parents before enrolling their children in the study. Parents and children were informed of their right to withdraw from the study at any time. The findings of “Proyecto Guagua” will be disseminated through open-access, peer-reviewed publications and presented at local and international scientific events.

## 1 Introduction

The gut microbiota (GM) plays a crucial role in maintaining human health because of its involvement in basic biological functions. The GM is required for the development and maturation of the human host’s intestinal epithelium and immune system. Likewise, it facilitates the host metabolism and adiposity by expanding nutrient sources, and producing vitamins (e.g., B-group vitamins and vitamin K) (1). Thanks to the GM, its human host is able to have access to short-chain fatty acid (SCFAs) that are the end-products of the fermentation of dietary fiber by the colonic microbiota and which play important roles in metabolism, immune response and inflammation (2). Alterations in the GM have been described in various conditions including infectious diseases, malnutrition (e.g., overweight, obesity, undernutrition), and neurodevelopmental disorders; however, not much is known about the specific role of the GM in these conditions (3–5).

Intestinal parasitic infections (IPI) and malnutrition are among the most frequent causes of illness in children from low and middle-income countries, who alike often suffer the consequences of poor basic sanitation, food insecurity and limited access to health services (6–8). Children infected with helminths (e.g., hookworms, *Ascaris lumbricoides*, *Trichuris trichiura*) are more likely to show underweight (sex-specific body mass index (BMI) for-age and weight for-age less than the 5th percentile), stunting (sex-specific height for-age less than the 5th percentile), and wasting (sex-specific weight for-height less than the 5th percentile) than their peers (9, 10). Stunting is also frequently observed among age-school children infected with protoza including *Giardia* and *Entamoeba* (11, 12).

Children suffering IPI and/or undernutrition (i.e., underweight, stunting and wasting) in general are more liable to show delays in the achievement of developmental milestones and neurocognitive difficulties than their peers (13–17). Neurocognitive difficulties in children suffering IPI and/or undernutrition might be the consequence of a sub-optimal brain development, which may be driven by reduced availability of nutrients, immuno-inflammatory mechanisms along with impaired barrier function that might allow harmful substances to infiltrate (18, 19). As far as the GM is tightly involved in these and other important biological functions it is important to understand better its contribution to the presence of neurocognitive difficulties in children suffering IPI and/or undernutrition (20).

Although IPI and malnutrition are both highly prevalent in Ecuadorian children, little is known on their association with neurocognitive difficulties or changes in the microbiota population structure, diversity or function (10, 21). Most of previous studies have focused only on malnutrition or parasitosis and restricted their target population to toddlers or children with neurodevelopmental disorders like autism spectrum disorder (ASD) (14, 16, 22–24). However, unlike those studies, the Proyecto Guagua takes a broader approach by critically and systematically studying IPI, malnutrition and neurocognitive functioning in Ecuadorian school-age children of the general population. The insights from the Proyecto Guagua will provide the basis for developing integral approaches to prevent IPI and malnutrition and their detrimental consequences on children’s health.

## 2 Methods and analyses

### 2.1 Study design and setting

**Figure 1:**
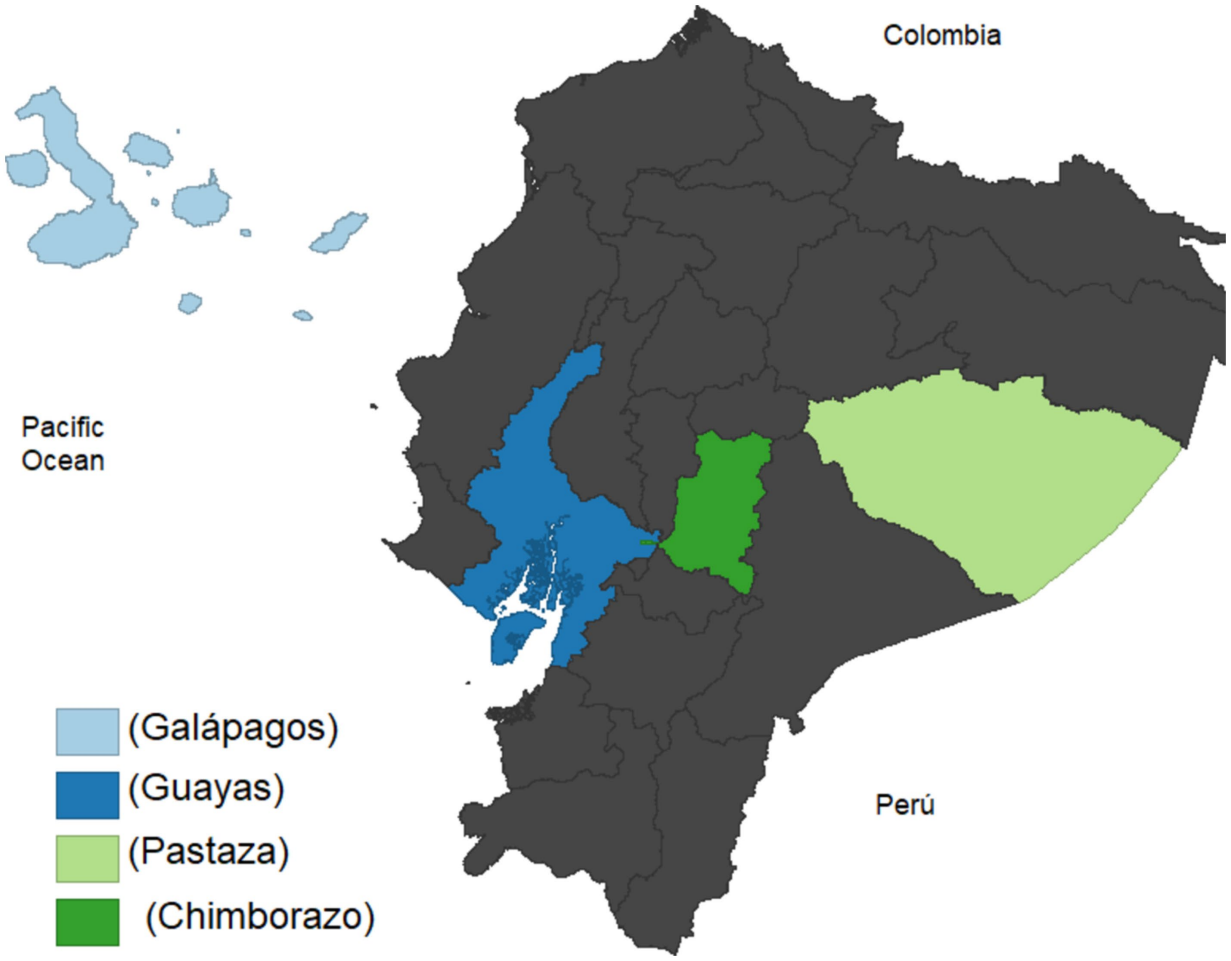
Geographical distribution of the sample population. The location of the schools that participate in the study is highlighted in different tones of blue and green.

The present is the protocol for a cross-sectional study called Proyecto Guagua, which translated from Ecuadorian Spanish to English means “project for the children.” The Proyecto Guagua is being carried out in various rural and urban counties distributed in each of the four geographical regions of Ecuador: the Pacific Coast, the Highlands, the Amazon, and the Galápagos Islands (Figure 1). The Proyecto Guagua started in 2022 and is still ongoing.

### 2.2 Ethical considerations

The research protocol of Proyecto Guagua was approved by the Research Ethics Committee of the Universidad de Cuenca in July 2022 (code: 2022-002EO-I). After getting the approval from the Research Ethics Committee, we conducted a pilot study that included 30 children to evaluate the procedures and instruments. Both instruments and procedures were then revised and adjusted accordingly. An edited protocol with minor changes was submitted to the Research Ethics Committee of the Universidad de Cuenca for approval of the changes. Before being included in the study, the parents and/or main caregivers are fully informed about the objective of the study, procedures, the data that is going to be collected, and what will be the data use for.

Parents and/or main caregivers should provide a signed written consent to participate in the study. They are explained that their participation is completely voluntary and that (i) not agreeing to participate will not have any consequence; (ii) they have the right to leave the study at any moment; (iii) they have the right to request the researchers not to use the information they provide or that of their children at any time; and (iv) the information they provide and that of their children will be used to write a report that will be publicly available; however, the report will not contain in any case personal information that allows their identification. Similarly, children are informed about the procedures before they are carried out, and that they will only be performed if they (the children) agree on being assessed, even if their parents have provided signed the consent.

### 2.3 Target population and sample

The Proyecto Guagua will recruit 450 school-age children between 6 and 12 years old, enrolled at public and private schools located in urban and rural counties in Ecuador (Supplementary Table 1). Non-probability sampling was applied to recruit participants. The sample size was calculated based on the results of a previous study that showed an association between IPI and changes in the gut microbiota (effect size, 0.72; mean difference, 70; standard deviation (SD), 96) (25). The sample size calculation indicated that we need 34 children with IPI and 34 without IPI (*β*, 0.80 and *α*, 0.05). Given the varied prevalence of IPI in Ecuador (ranging from 15% to 25%) we need to screen 189 children to identify a sufficient number of children with IPI (10). Nearly 30% of school-age children in Ecuador show malnutrition and this number is likely to be 20% higher in children with IPI (10). Therefore, we need 189 times 2 equal 378 to achieve the minimum number of children (*n* = 34) with IPI and malnutrition. No previous studies have been carried out to evaluate sensory motor integration and eye-blink conditioning in school-age children. Therefore we estimated the sample size for an estimated standardized effect size of 0.56 (β, 0.80 and α, 0.05) = 45 per group following the recommendations and Table 6A from (26). Standardized effect size of 0.56 corresponds to the expected effect size (% of conditional responses, 15) divided by the standard deviation from a study carried out in children without malnutrition (SD, 26) (27). Considering that the prevalence of IPI and malnutrition is expected to vary by area of residence (rural vs. urban) and geographical region, we decided to set the sample size to 450.

#### 2.3.1 Inclusion and exclusion criteria

Proyecto Guagua recruits (i) 6 to 12 years old female and male children attending primary school, (ii) who voluntarily agree to take part in the study, and (iii) whose parents provide written consent for them to participate. Exclusion criteria include: (i) Evident signs of acute disease such as fever; and (ii) evident signs of physical or cognitive disability.

### 2.4 Procedures

Potential participating schools and social organizations are first contacted through community representatives. Presentations about the project are held during parent-school and social organization meetings. Informational brochures were also distributed to introduce the study through media social networks in order to recruit participants for the pilot study. Interested families attend an informative session where eligibility is assessed based on the aforementioned criteria, and parents and/or legal guardians are informed about the nature of the study in detail. Consent forms are then provided to parents or legal guardians who decide to participate, ensuring they are fully informed about their participation rights and study procedures. The children are officially enrolled in the study upon obtaining the signed consent form and are then contacted to schedule the evaluation sessions.

During the evaluation sessions, parents or in their absence main caregivers (usually first-degree relatives) are asked to fill out a questionnaire with the guidance of trained field researchers. The questionnaire included several sections (Supplementary material 1): (i) Sociodemographic information; (ii) survey on household socioeconomic position; (iii) medical history and usage of medication; (iv) 24-h recall of food consumption; (v) Autism Spectrum Quotient 10-item inventory (AQ-10); (vi) Sleep Disturbance Scale for Children; and (vii) International Physical Activity Questionnaire for children (IPAQ-C).

Parents are also asked to bring a stool sample of their children next day. Parents and/or main caregivers are instructed by the field researchers on how and when to collect the samples and are provided with a recipient to do so. Field researchers also schedule meetings with parents and school teachers who voluntarily agree to participate in a face-to-face or Zoom interview to discuss about hygiene, nutrition, and management of foods. During their visits to the schools, field researchers fill out a form to collect information on water, sanitation, and hygiene (WASH) for each school that children attend. Trained field researchers assess the nutritional status of the children using anthropometry and bioimpedance. They also assess children’s Intelligence Quotient (IQ) using Raven’s Progressive Matrices as well as motor learning and sensory integration using a smartphone-based application called BlinkLab (28).

#### 2.4.1 Stool sample collection

Parents or main caregivers are provided with a labeled stool collection container and instructed on how to collect the sample from their children shortly before delivering it to the field researchers. After receiving the stool sample, field researchers aliquot the sample *in situ* into five aliquots to perform different analyses. The aliquots are kept at a portable cooler for transportation. The first aliquot is stored in special tubes (OMNIgene.Gut^®^ gut|OMR-200 or DANASTOOL Sample Collection MICROBIOME Kit^®^) and then stored at −80°C. The other four aliquots are fixed at 70% ethanol (two aliquots) or 90% ethanol (two aliquots) and stored at −80°C until they are analyzed. The first aliquot will be used to study the GM and the alcohol-fixed samples to examine the presence of intestinal parasites. One of the two 70% and one of the two 90% alcohol-fixed aliquots will be kept for future analyses.

### 2.5 Assessments

#### 2.5.1 Survey on household socioeconomic position

Household socioeconomic position of the participating families is evaluated using an Ecuadorian survey developed by the *Instituto Nacional de Estadísticas y Censos de Ecuador* (INEC) (29). The questionnaire has been previously nationally validated and widely used by the Ecuadorian government since 2010. The instrument consists on various closed-ended questions whose responses are rated with specific weights (29). It evaluates six dimensions to determine the socioeconomic position of the household: (i) Housing characteristics; (ii) access to technology; (iii) possession of material goods; (iv) consumption habits; (v) level of education of the head of the household; and (vi) household income. The final score corresponds to the sum of the values given to the answers and can range from 0 to 1,000 points, the higher the score the higher the socioeconomic position. Based on the score, households are classified into five groups of socioeconomic position: A (>845), high; B (>696 and ≤ 845), upper-middle; C+ (>535 and ≤ 696), middle; C− (>316 and ≤535), lower-middle; and D (≤316), low.

#### 2.5.2 Medical history and usage of medication

In this section of the questionnaire, the parents or main caregivers are asked about the general health and behavior of their children as well as access to health services using closed-ended and open-ended questions (Supplementary material 1). Likewise, they are asked about the use of antibiotics, antivirals, antiparasitic medication and other medications or treatments in the past month and year.

#### 2.5.3 Evaluation of diet and nutritional status

##### 2.5.3.1 24-h recall of food consumption

Data on the children’s recent food and beverages intake is collected using a 24-h dietary recall by trained field researchers (30). The 24-h recall records data on the following: (i) Time of the meal consumption, (ii) type and amount of food, and (iii) cooking method. Field researchers use the Ecuadorian photographic manual of portions to quantify food consumption (31). Upon data collection, a list of foods with their net weight in grams (g) or milliliters (mL) is prepared for each child. The nutritional composition of the meal is then estimated using the Ecuadorian and the Central American tables of nutritional composition of foods (32, 33). The amount of caloric intake measured in kilo-calories (kcal), as well as that of carbohydrates, proteins, fats and micronutrients is then estimated in g, milligrams (mg), or micrograms (μg) as appropriate. We will estimate the amounts of the following micronutrients: Vitamin A, calcium, phosphorus, iron, and sodium. These micronutrients were chosen because their deficit has been frequently associated with malnutrition in children. Data obtained from dietary intake will be then compared with the nutritional requirements of children to estimate the percentage of nutrient intake adequacy (34).

##### 2.5.3.2 Anthropometric measurements and bioimpedance

Anthropometric data is collected in an appropriate environment by trained field researchers following internationally standardized techniques (35–37). During the assessment children are barefoot and wear light clothing. All measurements are taken three times and then averaged. Height (measured in centimeters (cm)) is assessed using a Leicester^®^ stadiometer (accuracy, millimetre (mm)) and weight and body composition using an electronic digital scale (inBody^®^) for pediatric population (accuracy, 100 grams (gr)). Neck circumference (measured in cm) was measured using a non-stretchable measuring tape to the nearest 0.10 cm. The tape is placed on the midline of the neck between the cervical backbone and the anterior neck while the children stood upright, face forward, and shoulders relaxed. Waist circumference (measured in cm) is measured at the midpoint between the lowest border of the rib cage and the upper iliac crest to the nearest 0.1 cm. Arm circumference is measured at a mark point in the middle of the posterior surface of the left humerus between the acromion and the olecranon process.

Z-scores for weight/height, height/age, weight/age, and BMI/age are then calculated using the software World Health Organization (WHO) AnthroPlus (38). AnthroPlus uses the WHO Reference 2007 for 5–19 years to monitor the growth of school-age children and adolescents (39). Malnutrition was determined using the following indicators: stunting (height/age < −2 standard deviation (SD)); underweight (BMI/age or weight/age < −2 SD); wasting (weight/height < −2 SD); and overweight/obesity (BMI/age > +2 SD) (39). We will also apply Nutrimetry that uses height/age z scores and BMI/age z scores to produce nine groups of nutritional status and facilitate a combined interpretation of both indicators (40, 41). The cut-off points to determine the presence of malnutrition using arm circumference, neck circumference and waist circumference will be defined based on (37, 42, 43), respectively. The following indicators of body composition are analysed: (i) Total body fat percentage, (ii) visceral fat percentage, (iii) body water measured in liters, and (iv) muscle mass percentage.

#### 2.5.4 Sleep disturbance scale for children

Bruni’s Sleep Disturbance Scale for Children (SDSC) evaluates sleep quality and comprises 26 questions (44). The first two are multiple-choice and the rest Likert-type questions with a scale from 1 (never) to 5 (always) (Supplementary material 1). The questions are organized into sections that evaluate specific sleep disturbance categories (Table 1). The total score corresponds to the sum of the six sleep-disorder categories sub-scores, and ranges from 26 to 130. Higher scores indicate higher risk of suffering acute sleep disturbances, and a score equal or higher than 39 warrants further medical evaluation.

**Table 1:** Partial subscales of sleep problems according to the Bruni’s Sleep Disturbance Scale for Children (SDSC).

Partial subscales	Range
*Sleep onset and maintenance* Questions 1, 2, 3, 4, 5, 10, 11	7–35
*Respiratory problems* Questions 13, 14, 15	3–15
*Arousal disorders* Questions 17, 20, 21	3–15
*Disturbances of wake/sleep transition* Questions 6, 7, 8, 12, 18, 19	6–30
*Excessive drowsiness* Questions 22, 23, 24, 25, 26	5–25
*Sleep hyperhidrosis* Questions 9, 16	2–10

#### 2.5.5 International physical activity questionnaire for children (IPAQ-C)

This questionnaire comprises seven questions that measure the frequency, duration, and intensity of physical activity over the past 7 days and the time spent walking and sitting during a workday (45). Weekly physical activity is recorded in METs (Metabolic Equivalent of Task) per minute/week, using the following reference values: 3.3 METs for walking, 4 METs for moderate physical activity, and 8 METs for intense physical activity. The level of physical activity is calculated by multiplying METs values by the time spent in minutes per day and the number of days per week during which physical activity is performed. Based on this result, three levels of physical activity are defined (low, moderate, and high) (Table 2).

**Table 2:** Physical activity level is assessed using the International Physical Activity Questionnaire for children (IPAQ-C).

Low (Category 1)	No physical activity
	Not enough physical activity of at least 600 meters per min/week.
Moderate (Category 2)	Three or more days of vigorous physical activity for at least 25 min per day.
	Five or more days of moderate physical activity and/or walking at least 30 min per day.
	Five or more days of walking, moderate and/or intense physical activity of at least 600 meters per min/week.
High (Category 3)	At least 3 days per week of intense physical activity of 1,500 meters per min/week.
	Seven or more days per week of walking, moderate and/or intense physical activity of at least 3,000 METs per min/week.

#### 2.5.6 Cognitive and neurobehavioral assessment

##### 2.5.6.1 Cognitive ability assessment

General cognitive ability is assessed using the Raven’s Progressive Matrices (41). Raven’s Progressive Matrices is a non-verbal test that consists of 36 multiple choice questions, which are listed in order of increasing difficulty. During the test the children are asked to identify the missing element that completes the pattern. The test results will be expressed in terms of Intelligence Quotient (IQ).

##### 2.5.6.2 Social interaction skills

Social interaction skills are assessed using the AQ-10 questionnaire (children’s version). This questionnaire is designed to be brief (3–5 min), easy to use, and self-administered. It consists of 10 questions with four possible responses: (i) Strongly agree (scored as 0); (ii) partially agree (scored as 1); (iii) partially disagree (scored as 2); and (iv) strongly disagree (scored as 3) (46, 47). The wording of the questions was adapted to Ecuadorian Spanish (Supplementary material 1). The score of the instrument corresponds to the sum of all questions and can range from 0 to 30 points. A score higher than 6 suggests difficulties in social interaction and warrants further medical evaluation.

##### 2.5.6.3 Sensory motor integration and motor learning

Sensory motor integration and motor learning evaluation are conducted using BlinkLab, a smartphone-based application that allows the non-invasive administration of neurometric tests. These include the acoustically evoked eyelid startle reflex, pre-pulse inhibition, eye-blink conditioning, and startle habituation (28). During the experiment, children watch an entertaining movie while the trials containing the auditory stimuli are delivered via headphones. For each trial, computer vision algorithms are used to track and record the position of the participant’s facial landmarks over time. The sessions are conducted in a quiet environment, free from distractions such as noise or bright lights, and take between 15 and 20 min to complete. The test is administered three times within a week.

The individual eye-blink traces will be analyzed with custom computer software using R. Eyelid position will be calculated based on the facial landmarks. Eye-blink traces are filtered forward and reverse with a low-pass Butterworth filter. Trials with significant activity in the pre-conditioning stimulus (CS) period [> 7 times the interquartile range (IQR)] are regarded as invalid and disregarded for further analysis. Trials are min-max normalized by aligning the pre-CS baselines and normalizing the signal, so that the size of a full blink is one normalized eye closure (NEC). This normalization is achieved by using the reflexive blinks in the unconditioned stimulus (US) only trials as a reference. For each session, we calculate the maximum value in the averaged unconditioned response (UR), and individual traces are normalized by dividing each trace by this value. Consequently, in the normalized traces, an NEC of 1 corresponded with the eye being fully closed, and an NEC of 0 corresponded with the eye being fully open.

In valid normalized trials, we will determine the following four outcome measures: (i) *Amplitude of a conditional response (CR):* The normalized eye closure amplitude, which measures the strength of the response. A larger response means a better CR. (ii) *Latency to CR onset:* The first deviation from baseline level, which is a measure of the adaptive timing or accuracy of the CR. An eyelid closure that starts late in the CS-US interval indicates better timing. (iii) *Latency to CR peak*: The peak of the eye-blink CR, which is a measure of the adaptive timing or accuracy of the CR. The optimal CR is where the eyelid is maximally closed at US delivery. (iv) *Presence of a CR (CR proportion)*: A CR is defined as all eyelid movements larger than a predefined threshold (for instance >0.1 NEC) with latency to CR onset and latency to CR peak between predefined intervals.

Based on these four outcome measures, we can study phenomena like acquisition, savings and retention, extinction, generalization, latent inhibition, over-expectation, etc. We will study both learning speed (how fast the subject learns the task) and learning accuracy (how well the timing of eye-blink CRs is). We will include both paired CS-US and CS-only trials. CS-only trials show the full kinetic profile of the eye-blink CR, providing better information about the adaptive timing of eye-blink CRs.

#### 2.5.7 Interviews

To explore perceptions about knowledge and practices regarding hygiene, nutrition, and food handling, trained field researchers interview a sub-sample of parents or main caregivers and teachers. The interview is thematic and semi-structured, and takes between 30 min and one hour. We extend an invitation to all parents or main caregivers and teachers in all the schools that participate in the proyecto GUAGUA. Participation in the interview is self-selected and we will recruit participants until data saturation (i.e., repetition, absence of novelty) is reached. We schedule face-to-face or Zoom interviews with interested participants, depending on their availability. The interview is recorded in order to be first transcribed and then analyzed. Interviews’ thematic analysis will be carried out with the help of the software Atlas ti v 24.1.1.30813 and using an inductive approach (48). The analysis will include six steps: (i) familiarization with the data; (ii) creation of initial codes; (iii) searching themes in the data; (iv) reviewing the themes; (v) defining and naming the themes; and (vi) writing the report (49). The thematic analysis will be also presented as thematic networks (50).

#### 2.5.8 Field observation of schools

Field researchers collected information on drinking-water and sanitation facilities of schools using an observation form (WASH) that was developed based on (51). In each school, field researchers gather information from school representatives and visited the school facilities while filling out the observation form (Supplementary material 2).

#### 2.5.9 Detection of intestinal parasite infection

There is no gold standard to detect intestinal parasites, and therefore we decided to combine the results of various methods to increase sensitivity and specificity. Two techniques will be applied to detect the presence of parasites in the stool samples: (i) light microscopy (i.e., Richie’s method) using 70% alcohol-fixed aliquots, and molecular identification of *Strongyloides stercoralis* and *Entamoeba histolytica/dispar* using 90% alcohol-fixed aliquots. Ritchie’s method, which is relatively cheap and simple to implement, performs better than other similar methods for the identification of pathogenic and commensal protozoa (52). Compared to the combined results of wet mount, modified Baermann’s and Ritchie’s method, the last has shown 68% [95% confidence intervals (CI), 59: 76] of sensitivity, and 100% [95% CI, 96: 100] of specificity to detect IPI (53). Real-time polymerase chain reaction (PCR) will be used for molecular detection of *Strongyloides stercoralis* and *Entamoeba histolytica/dispar*, following the protocols from (54, 55). It has been reported that PCR has a sensitivity of 60% [95% CI, 55: 67] and specificity of 99% [95% CI, 99: 100] for detecting *Strongyloides stercoralis* (55). PCR has been found to be more sensitive than microscopy and culture (88%) and 100% specific for detecting each of the two *Entamoeba* species (56).

#### 2.5.10 Gut microbiota analysis

Marker gene amplification and sequencing, which uses primers that target a specific region of a gene of interest in order to determine phylogenies of a sample, will be used to characterize the microbial composition of the children GM. This approach works well for samples contaminated by host deoxyribonucleic acid (DNA), such as tissue and low-biomass samples (57). Previous studies have shown that this approach is both precise in determining the taxonomic microbial profile and accurate for diversity analysis (58). The 16S ribosomal ribonucleic acid (rRNA) gene, which contains a highly variable region that is used for detailed identification (i.e., V4 region), will be used as marker gene. V4 is flanked by highly conserved regions that can serve as binding sites for primers during PCR amplification (57). Details on how marker gene amplification and sequencing will be performed can be found in Supplementary material 3. Briefly, first total genomic DNA (gDNA) will be isolated from the stool samples using a bead-beating-based purification method. Second, the V4 hypervariable region of the 16S rRNA gene will be amplified using i5/i7 barcoded primers and sequenced in a 2 × 300 base pair (bp) paired-end run with the aid of the platform Illumina MiSeq^®^. Stool and gDNA sample remains will be properly stored at −80°C for future metagenomic analyses.

Sequenced reads will be processed using QIIME 22024.2 and then clustered into amplicon sequence variants (ASVs) using the DADA2 pipeline. The taxonomic classification of the sequenced 16S rRNA V4 region will be determined using the Greengenes database as a reference (59). The similarity between microbiomes of the groups will be visualized with Principal Coordinates Analysis (PCoA) of ASVs based on Bray–Curtis dissimilarity (59). Differences in microbiome composition (beta-diversity) among groups of children belonging to different categories of nutritional or IPI status will be assessed by Bray–Curtis dissimilarity using the ADONIS2 implementation of permutational multivariate analysis of variance (PERMANOVA) in vegan: Community Ecology Package. Homogeneity of variance will be tested for each group using betadisper in vegan. Differences in ASV abundance and diversity across different groups of children will be calculated using the pairwise Wilcoxon rank sum test with *p*-value adjustment by Benjamini-Hochberg correction for multiple comparisons (59).

### 2.6 Statistics analysis

Initial analyses will involve descriptive statistics to summarize the data, including means, standard deviations, medians, interquartile ranges for continuous variables, and frequencies and percentages for categorical variables. Bivariate analyses will examine the relationships between key variables such as parasitic infections and changes in microbiota diversity, nutritional status, and developmental outcomes.

Before inferential analyses, assumptions of normality and homoscedasticity will be assessed. Residual plots will be examined for any apparent deviations from randomness, indicating potential violations of these assumptions. The Shapiro–Wilk test will assess normality, and the Breusch-Pagan test will be employed to check for homoscedasticity. In cases where assumptions of normality and homoscedasticity are not met, we will opt for non-parametric methods such as the Mann–Whitney U test or the Kruskal-Wallis test when comparing two or more groups. Bootstrapping methods will be employed for more complex models to provide robust standard errors and confidence intervals that are not dependent on normality assumptions.

Multivariate regression models will be used to adjust for potential confounders, including age, sex, socioeconomic status, and region. These models will help determine the independent effects of parasitic infections and microbiota on nutritional and neurocognitive outcomes. Logistic regression will be employed for binary outcomes (e.g., presence or absence of specific parasitic infections), while linear regression will be used for continuous outcomes (e.g., neurocognitive scores). Mixed-effects models will be utilized for variables measured repeatedly over the study period (e.g., eye blink conditioning). These models account for the correlation of repeated measures within the same subjects and allow for the analysis of fixed effects (e.g., sex) and random effects (e.g., individual intercepts for learning curves).

All analyses will be conducted using R statistical software. The STROBE guidelines will be applied to report the results. Measures of effect will be presented with 95% confidence intervals, and all tests will be two-sided with a significance level set at *p* < 0.05.

## 3 Discussion

The present is the protocol of the Proyecto Guagua, a cross-sectional study that is being carried out in Ecuador with the financial support of the Escuela Superior Politécnica de Chimborazo. The study aims to describe differences in microbiota population structure and diversity between (i) school-age children with malnutrition (e.g., undernourished and obese/overweight children) and their peers; (ii) school-age children with parasitosis and their peers. Proyecto Guagua aims at testing the hypothesis that an altered GM mediates the influence of malnutrition on neurocognitive functioning and parasitosis status. The insights from the Proyecto Guagua will provide a theoretical basis for developing integral public health interventions to prevent and/or treat malnutrition and IPI as well as their consequences on children’s health.

Similar studies than Proyecto Guagua have mainly focused in under 5 years of age children with severe nutritional conditions or with neurodevelopmental disorders. For example, bacterial population shifts, globally decreased diversity, and enrichment of potentially pathogenic bacteria have been described in the GM of children under 5 years of age with wasting or kwashiorkor in comparison to peers without malnutrition (60, 61). Bacterial population shifts have also been observed in the GM of children under 5 years of age suffering parasitosis (22, 25). It is likely that some of these differences are explained by diet, previous use of medication or other socio-demographic factors such as biological sex, age, and socioeconomic position (62). The influence of such factors has not been systematically studied regarding bacterial population shifts in children with malnutrition and/or parasitosis and that is the gap that Proyecto Guagua tries to fill up.

Nutrition during critical phases of development has long-term effects on organ size, structure and function and as such it is believed to play an important role on the development of the child’s brain (63). Nutrition is also involved in adaptive and innate immunity, which is essential for defense against parasitic infections (64). The GM, which interacts closely with host diet, has the capacity to modulate brain function and behavior, and therefore could mediate part of the adverse effects of malnutrition on neurocognitive functioning (19). The GM could also mediate the detrimental effects of malnutrition on the host capacity to resist or overcome the effects of IPI (65). The GM might influence neurocognitive functioning and susceptibility to IPI by modulating the host metabolism, biochemical signaling, immune function, stress-responsivity, and epigenetic regulation (1, 2). Proyecto Guagua will allow us to test these hypotheses.

Nevertheless, Proyecto Guagua has several limitations that must be considered when interpreting future results. First, the design is cross-sectional and therefore warrants against any causal interpretation. The idea of Proyecto Guagua at this stage is to reproduce previous findings in school-age children, taking into account the influence of diet, previous use of medication or other socio-demographic factors as well as opening new lines of research. Second, sample selection is not probabilistic and therefore the results of Proyecto Guagua are likely affected by selection bias. Representativeness, however, it does not seem essential to study exposure-outcome associations, which is the aim of Proyecto Guagua (66).

A third limitation might arise from the precision and accuracy of the instruments and techniques which Proyecto Guagua is using to collect the information. To improve the precision and accuracy we (i) standardize the measured methods in an operational manual; (ii) train field researchers, (iii) whenever possible use automatic measure devices (e.g., inBody^®^, BlinkLab), and (iv) use the mean of three anthropometric measures (e.g., height, weight, neck, waist and arm circumferences). To assure validity, we used instruments that have been previously validated (e.g., Survey on household socioeconomic position, 24-h recall, AQ-10, SDSC, IPAQ-C, Raven’s Progressive Matrices).

## References

[1] Sommer F Bäckhed F. The gut microbiota — masters of host development and physiology. Nat Rev Microbiol. (2013) 11:227–38. doi: 10.1038/nrmicro2974, PMID: 23435359

[2] Tremaroli V Bäckhed F. Functional interactions between the gut microbiota and host metabolism. Nature. (2012) 489:242–9. doi: 10.1038/nature11552, PMID: 22972297

[3] Fang Y Lei Z Zhang L Liu CH Chai Q. Regulatory functions and mechanisms of human microbiota in infectious diseases. hLife. (2024) 2:496–513. doi: 10.1016/j.hlife.2024.03.004

[4] Borre YE O’Keeffe GW Clarke G Stanton C Dinan TG Cryan JF. Microbiota and neurodevelopmental windows: implications for brain disorders. Trends Mol Med. (2014) 20:509–18. doi: 10.1016/j.molmed.2014.05.002, PMID: 24956966

[5] Zoghi S Sadeghpour F Nikniaz Z Shirmohamadi M Moaddab SY Ebrahimzadeh LH. Gut microbiota and childhood malnutrition: understanding the link and exploring therapeutic interventions. Eng Life Sci. (2024) 24:2300070. doi: 10.1002/elsc.202300070, PMID: 38708416 PMC11065333

[6] Hotez PJ Bottazzi ME Franco-Paredes C Ault SK Periago MR. The neglected tropical diseases of Latin America and the Caribbean: a review of disease burden and distribution and a roadmap for control and elimination. PLoS Negl Trop Dis. (2008) 2:e300. doi: 10.1371/journal.pntd.0000300, PMID: 18820747 PMC2553488

[7] Barquera S Oviedo C Buenrostro N White M. The double burden of malnutrition in Latin America. United Nations Expert Group Meeting on population, food security, nutrition and sustainable development for sustainable development. United Nations Secretariat (2019).

[8] Barreto SM Miranda JJ Figueroa JP Schmidt MI Munoz S Kuri-Morales PP . Epidemiology in Latin America and the Caribbean: current situation and challenges. Int J Epidemiol. (2012) 41:557–71. doi: 10.1093/ije/dys017, PMID: 22407860 PMC3324459

[9] Opara KN Udoidung NI Opara DC Okon OE Edosomwan EU Udoh AJ. The impact of intestinal parasitic infections on the nutritional status of rural and urban school-aged children in Nigeria. Int J MCH AIDS. (2012) 1:73–82. doi: 10.21106/ijma.8, PMID: 27621960 PMC4948163

[10] Moncayo AL Lovato R Cooper PJ. Soil-transmitted helminth infections and nutritional status in Ecuador: findings from a national survey and implications for control strategies. BMJ Open. (2018) 8:e021319. doi: 10.1136/bmjopen-2017-021319, PMID: 29705768 PMC5931300

[11] Sackey ME Weigel MM Armijos RX. Predictors and nutritional consequences of intestinal parasitic infections in rural Ecuadorian children. J Trop Pediatr. (2003) 49:17–23. doi: 10.1093/tropej/49.1.17, PMID: 12630715

[12] Petri WA Jr Mondal D Peterson KM Duggal P Haque R. Association of malnutrition with amebiasis. Nutr Rev. (2009) 67:S207–15. doi: 10.1111/j.1753-4887.2009.00242.x, PMID: 19906225

[13] Benton D. The influence of children’s diet on their cognition and behavior. Eur J Nutr. (2008) 47:25–37. doi: 10.1007/s00394-008-3003-x, PMID: 18683027

[14] Huiracocha-Tutiven L Orellana-Paucar A Abril-Ulloa V Huiracocha-Tutiven M Palacios-Santana G Blume S. Child development and nutritional status in Ecuador. Global Pediatric Health. (2019) 6:1–12. doi: 10.1177/2333794X18821946, PMID: 30719492 PMC6348541

[15] Zulkarnaen Z. The influence of nutritional status on gross and fine motor skills development in early childhood. Asian Soc Sci. (2019) 15:75. doi: 10.5539/ass.v15n5p75

[16] Cavagnari BM Guerrero-Vaca DJ Carpio-Arias TV Duran-Aguero S Vinueza-Veloz AF Robalino-Valdivieso MP . The double burden of malnutrition and gross motor development in infants: a cross-sectional study. Clin Nutr. (2023) 42:1181–8. doi: 10.1016/j.clnu.2023.05.001, PMID: 37225559

[17] Suryawan A Jalaludin MY Poh BK Sanusi R Tan VMH Geurts JM . Malnutrition in early life and its neurodevelopmental and cognitive consequences: a scoping review. Nutr Res Rev. (2022) 35:136–49. doi: 10.1017/S0954422421000159, PMID: 34100353

[18] Amaruddin AI Koopman JPR Muhammad M Lenaerts K van Eijk HMH Brienen EAT . Intestinal permeability before and after albendazole treatment in low and high socioeconomic status schoolchildren in Makassar, Indonesia. Sci Rep. (2022) 12:3394. doi: 10.1038/s41598-022-07086-7, PMID: 35233023 PMC8888571

[19] Coley EJL Hsiao EY. Malnutrition and the microbiome as modifiers of early neurodevelopment. Trends Neurosci. (2021) 44:753–64. doi: 10.1016/j.tins.2021.06.004, PMID: 34303552

[20] Warner BB. The contribution of the gut microbiome to neurodevelopment and neuropsychiatric disorders. Pediatr Res. (2019) 85:216–24. doi: 10.1038/s41390-018-0191-9, PMID: 30283047

[21] Hajri T Angamarca-Armijos V Caceres L. Prevalence of stunting and obesity in Ecuador: a systematic review. Public Health Nutr. (2021) 24:2259–72. 32723419 10.1017/S1368980020002049 PMC10195486

[22] Zurita MF Cárdenas PA Sandoval ME Peña MC Fornasini M Flores N . Analysis of gut microbiome, nutrition and immune status in autism spectrum disorder: a case-control study in Ecuador. Gut Microbes. (2020) 11:453–64. doi: 10.1080/19490976.2019.1662260, PMID: 31530087 PMC7524316

[23] Cooper P Walker AW Reyes J Chico M Salter SJ Vaca M . Patent human infections with the whipworm, Trichuris trichiura, are not associated with alterations in the faecal microbiota. PLoS One. (2013) 8:e76573. doi: 10.1371/journal.pone.0076573, PMID: 24124574 PMC3790696

[24] Soto-Girón MJ Peña-Gonzalez A Hatt JK Montero L Páez M Ortega E . Gut microbiome changes with acute diarrheal disease in urban versus rural settings in northern Ecuador. Am J Trop Med Hyg. (2021) 104:2275–85. doi: 10.4269/ajtmh.20-0831, PMID: 33872206 PMC8176484

[25] Toro-Londono MA Bedoya-Urrego K Garcia-Montoya GM Galvan-Diaz AL Alzate JF. Intestinal parasitic infection alters bacterial gut microbiota in children. PeerJ. (2019) 7:e6200. doi: 10.7717/peerj.6200, PMID: 30643702 PMC6327884

[26] Hulley SB Cummings SR Browner WS Grady DG Newman TB. Designing clinical research. Philadelphia, USA: Lippincott Williams & Wilkins (2013). 611 p.

[27] Löwgren K Bååth R Rasmussen A Boele HJ Koekkoek SKE de C . Performance in eyeblink conditioning is age and sex dependent. PLoS One. (2017) 12:e0177849. doi: 10.1371/journal.pone.0177849, PMID: 28542383 PMC5436819

[28] Boele HJ Jung C Sherry S Roggeveen LEM Dijkhuizen S Öhman J . Accessible and reliable neurometric testing in humans using a smartphone platform. Sci Rep. (2023) 13:22871. doi: 10.1038/s41598-023-49568-2, PMID: 38129487 PMC10739701

[29] Instituto Nacional de Estadística y Censos. Instituto Nacional de Estadística y Censos. Encuesta de Estratificación del Nivel Socioeconómico. Available at: https://www.ecuadorencifras.gob.ec/encuesta-de-estratificacion-del-nivel-socioeconomico/

[30] Ferrari MA. Estimación de la Ingesta por Recordatorio de 24 Horas. Diaeta. (2013) 31:20–5.

[31] Fontana MEH Chisaguano M Vayas G Crispim SP. Manual fotográfico de porciones para cuantificación alimentaria del Ecuador. 2.a ed. Quito, Ecuador: USFQ PRESS (2022).

[32] Ortiz Ulloa SJ Astudillo Rubio GC Donoso Moscoso SP Ochoa Aviles AM. Tabla de composición de alimentos Cuenca, Ecuador. Cuenca, Ecuador: Universidad de Cuenca (2018).

[33] Instituto de Nutrición de Centro América y Panamá (INCAP), Organización Panamericana de la Salud (OPS). Tabla de composición de alimentos de Centro América. 2nd ed. Guatemala: Organización Panamericana de la Salud (OPS) (2012).

[34] Hernández Triana M. Recomendaciones nutricionales para el ser humano: actualización. Revista Cubana de Investigaciones Biomédicas. (2004) 23:266–92.

[35] Lohman TG. Applicability of body composition techniques and constants for children and youths. Exerc Sport Sci Rev. (1986) 14:325–57. doi: 10.1249/00003677-198600140-00014, PMID: 3525188

[36] Cole TJ Bellizzi MC Flegal KM Dietz WH. Establishing a standard definition for child overweight and obesity worldwide: international survey. BMJ. (2000) 320:1240–3. doi: 10.1136/bmj.320.7244.1240, PMID: 10797032 PMC27365

[37] Fernández JR Redden DT Pietrobelli A Allison DB. Waist circumference percentiles in nationally representative samples of African-American, European-American, and Mexican-American children and adolescents. J Pediatr. (2004) 145:439–44. doi: 10.1016/j.jpeds.2004.06.044, PMID: 15480363

[38] World Health Organization. WHO Anthro Survey Analyser and other tools. (2023). Available at: https://www.who.int/tools/child-growth-standards/software

[39] Schumacher D. Anthro: computation of the WHO child growth standards. (2021). Available at: https://cran.r-project.org/web/packages/anthro/readme/README.html

[40] Tapia-Veloz E Gozalbo M Tapia-Veloz G Carpio-Arias TV Trelis M Guillén M. Evaluation of school children nutritional status in Ecuador using nutrimetry: a proposal of an education protocol to address the determinants of malnutrition. Nutrients. (2022) 14:3686. doi: 10.3390/nu14183686, PMID: 36145057 PMC9502477

[41] Selem-Solís JE Alcocer-Gamboa A Hattori-Hara M Esteve-Lanao J Larumbe-Zabala E. Nutrimetry: BMI assessment as a function of development. Endocrinol Diabetes Nutr. (2018) 65:84–91. doi: 10.1016/j.endinu.2017.10.009, PMID: 29276173

[42] Frisancho AR. Anthropometric standards: an interactive nutritional reference of body size and body composition for children and adults. 2nd ed. Ann Arbor: University of Michigan Press (2008).

[43] Filgueiras M Albuquerque F Castro A Rocha N Milagres L Novaes J. Neck circumference cutoff points to identify excess android fat. J Pediatr. (2020) 96:356–63. doi: 10.1016/j.jped.2018.11.009, PMID: 30731052 PMC9432153

[44] Bruni O Ottaviano S Guidetti V Romoli M Innocenzi M Cortesi F . The sleep disturbance scale for children (SDSC) construct ion and validation of an instrument to evaluate sleep disturbances in childhood and adolescence. J Sleep Res. (1996) 5:251–61. doi: 10.1111/j.1365-2869.1996.00251.x, PMID: 9065877

[45] Mantilla Toloza SC Gómez-Conesa A. El Cuestionario Internacional de Actividad Física. Un instrumento adecuado en el seguimiento de la actividad física poblacional. Revista Iberoamericana de Fisioterapia y Kinesiología. (2007) 10:48–52. doi: 10.1016/S1138-6045(07)73665-1

[46] Auyeung B Baron-Cohen S Wheelwright S Allison C. The autism spectrum quotient: children’s version (AQ-child). J Autism Dev Disord. (2008) 38:1230–40. doi: 10.1007/s10803-007-0504-z, PMID: 18064550

[47] Archibald T Willmott E Kelly C Bradbury L Hugo P Bryant-Waugh R. Investigating the utility of the AQ-10 in children and adolescents assessed in an outpatient ARFID clinic. Autism Res. (2024) 17:1867–75. doi: 10.1002/aur.3220, PMID: 39188093

[48] Thomas DR. A general inductive approach for analyzing qualitative evaluation data. Am J Eval. (2006) 27:237–46. doi: 10.1177/1098214005283748, PMID: 39790903

[49] Braun V Clarke V. Using thematic analysis in psychology. Qual Res Psychol. (2006) 3:77–101. doi: 10.1191/1478088706qp063oa

[50] Attride-Stirling J. Thematic networks: an analytic tool for qualitative research. Qual Res. (2001) 1:385–405. doi: 10.1177/146879410100100307

[51] World Health Organization. Core questions on drinking-water and sanitation for households surveys. (2026). Available at: https://www.who.int/publications/i/item/9241563265

[52] Navone G Gamboa M Kozubsky L Costas M Cardozo M Sisliauskas M . Estudio comparativo de recuperación de formas parasitarias por tres diferentes métodos de enriquecimiento coproparasitológico. Parasitol Latinoam. (2005) 60:178–81. doi: 10.4067/S0717-77122005000200014, PMID: 27315006

[53] Bayayibign B Melaku M Gelaye W. Performance evaluation of three laboratory diagnostic methods for intestinal parasitic infections at rural Bahir Dar, Northwest Ethiopia: a cross-sectional study. J Health Sci. (2019) 9:34–9. doi: 10.17532/jhsci.2019.170

[54] Guevara Á Vicuña Y Costales D Vivero S Anselmi M Bisoffi Z . Use of real-time polymerase chain reaction to differentiate between pathogenic Entamoeba histolytica and the nonpathogenic Entamoeba dispar in Ecuador. Am J Trop Med Hyg. (2019) 100:81–2. doi: 10.4269/ajtmh.17-1022, PMID: 30398142 PMC6335901

[55] Tamarozzi F Guevara ÁG Anselmi M Vicuña Y Prandi R Marquez M . Accuracy, acceptability, and feasibility of diagnostic tests for the screening of Strongyloides stercoralis in the field (ESTRELLA): a cross-sectional study in Ecuador. Lancet Glob Health. (2023) 11:e740–8. doi: 10.1016/S2214-109X(23)00108-0, PMID: 36972722

[56] Blessmann J Buss H Nu PAT Dinh BT Ngo QTV van A . Real-time PCR for detection and differentiation of Entamoeba histolytica and Entamoeba dispar in fecal samples. J Clin Microbiol. (2002) 40:4413–7. doi: 10.1128/JCM.40.12.4413-4417.2002, PMID: 12454128 PMC154634

[57] Knight R Vrbanac A Taylor BC Aksenov A Callewaert C Debelius J . Best practices for analysing microbiomes. Nat Rev Microbiol. (2018) 16:410–22. doi: 10.1038/s41579-018-0029-9, PMID: 29795328

[58] Aarnoutse R de Vos-Geelen JMPGM Penders J Boerma EG Warmerdam FARM Goorts B . Study protocol on the role of intestinal microbiota in colorectal cancer treatment: a pathway to personalized medicine 2.0. Int J Color Dis. (2017) 32:1077–84. doi: 10.1007/s00384-017-2819-3, PMID: 28444508 PMC5486633

[59] Henry LP Ayroles JF. *Drosophila melanogaster* microbiome is shaped by strict filtering and neutrality along a latitudinal cline. Mol Ecol. (2022) 31:5861–71. doi: 10.1111/mec.16692, PMID: 36094780 PMC9643648

[60] Monira S Nakamura S Gotoh K Izutsu K Watanabe H Alam NH . Gut microbiota of healthy and malnourished children in Bangladesh. Front Microbiol. (2011) 2:228. doi: 10.3389/fmicb.2011.00228, PMID: 22125551 PMC3221396

[61] Tidjani Alou M Million M Traore SI Mouelhi D Khelaifia S Bachar D . Gut bacteria missing in severe acute malnutrition, can we identify potential probiotics by culturomics? Front Microbiol. (2017) 8:899. doi: 10.3389/fmicb.2017.00899, PMID: 28588566 PMC5440526

[62] West KA Yin X Rutherford EM Wee B Choi J Chrisman BS . Multi-angle meta-analysis of the gut microbiome in autism Spectrum disorder: a step toward understanding patient subgroups. Sci Rep. (2022) 12:17034. doi: 10.1038/s41598-022-21327-9, PMID: 36220843 PMC9554176

[63] Cusick SE Georgieff MK. The role of nutrition in brain development: the Golden opportunity of the “first 1000 days”. J Pediatr. (2016) 175:16–21. doi: 10.1016/j.jpeds.2016.05.013, PMID: 27266965 PMC4981537

[64] Hughes S Kelly P. Interactions of malnutrition and immune impairment, with specific reference to immunity against parasites. Parasite Immunol. (2006) 28:577–88. doi: 10.1111/j.1365-3024.2006.00897.x, PMID: 17042929 PMC1636690

[65] Grondin JA Jamal A Mowna S Seto T Khan WI. Interaction between intestinal parasites and the gut microbiota: implications for the intestinal immune response and host defence. Pathogens. (2024) 13:608. doi: 10.3390/pathogens13080608, PMID: 39204209 PMC11356857

[66] Rothman KJ Gallacher JE Hatch EE. Why representativeness should be avoided. Int J Epidemiol. (2013) 42:1012–4. doi: 10.1093/ije/dys223, PMID: 24062287 PMC3888189

