# Supplementary material for: Microbiota, parasitic infections and their relationship with nutritional status and neurocognitive functioning in children from Ecuador—Proyecto Guagua: research protocol for a cross-sectional study
Source: Front Public Health. 2025 Jan 29;13:1505780. doi: 10.3389/fpubh.2025.1505780 (PMC11815663; doi:10.3389/fpubh.2025.1505780)
Supplement: Supplementary file 1 [file Data_Sheet_1.pdf]

1. ESTADO NUTRICIONAL DE LA NIÑA/O

CÓDIGO NIÑA/O: _____________

Nombres y apellidos de la niña/o:____

Nota: *Tomar los valores antropométricos* ***TRES veces****. Tomar las mediciones dos horas después de haber comido:*

Peso 1 en kg: _____ Peso 2 en kg:____ Peso 3 en kg:______

Talla 1 en metros: _____ Talla 2 en metros: _____ Talla 3 en metros: _____

a Antropometría:

- Circunferencia abdominal 1 en cm: _____ Circunferencia abdominal 2 en cm: _____

  Circunferencia abdominal 3 en cm: _____

- Circunferencia del brazo 1 en cm_____ Circunferencia del brazo 2 en cm_____

  Circunferencia del brazo 3 en cm_____

- Circunferencia del cuello 1 en cm_____ Circunferencia del cuello 2 en cm_____

  Circunferencia del cuello 3 en cm_____

b Bioimpedancia:

Dato de bioimpedancia (% que marque la balanza de bioimpedancia eléctrica):________

Nota: Verificar que el paciente haya orinado y no haya comido dos horas antes.

- Porcentaje de masa grasa:__________Porcentaje de grasa visceral:________
- Porcentaje de masa muscular:_______Minerales:________________

Observación:_________________________

2. EVALUACIÓN COGNITIVA Y CONDICIONAMIENTO DEL PARPADEO

CÓDIGO NIÑA/O: _____________

Nombres y apellidos de la niña/o:____

1. Se evalúa las funciones cognitivas a través del test RAVEN: Si ( ), No ( )
2. Fecha evaluación test Raven (dd/mm/aaa): __/__/____
3. Hora evaluación test Raven (hh:mm): __:__

4. Se evalúa las funciones cognitivas a través de blinklab: Si ( ), No ( )
5. Fecha evaluación test blinklab (dd/mm/aaa): __/__/____
6. Hora evaluación test blinklab (hh:mm): __:__

OBSERVACIONES:
____
____
____
____
____

3. ENCUESTA PARA EL REPRESENTANTE LEGAL DE LA NIÑA/O

CÓDIGO NIÑA/O: ____________

Nombres y apellidos de la niña/o:____

NOTA: *Este cuestionario será administrado por personal capacitado del proyecto vía telefónica o en persona, en la escuela o vivienda dependiendo de la decisión de la representante legal.*

Relación que tiene la representante legal con la niña/o: ☐ Madre ☐ Padre ☐ Otro_____

Nombres representante legal:_______________________

Apellidos representante legal:_______________________

Número teléfono celular representante legal: ______________

Correo electrónico representante legal:______________________

Dirección de la casa donde vive la niña/o: Calle principal:________________________________

Calle secundaria:____________________________ Localidad:_______________________

Cantón:______________________ Provincia_______________________

Edad de la **MADRE** (años):___________

Nivel de educación de la **MADRE** (marcar con una x según corresponda), incluya el número de años del último nivel alcanzado:

No sabe leer ni escribir___ Primaria incompleta___ Primaria completa___ Secundaria incompleta____ Secundaria completa___ Educación universitaria incompleta___ Educación universitaria completa____ Cuarto nivel___ Otro(Indique cual)_______

Número de años, último nivel educativo alcanzado:______

Edad del **PADRE** (años):___________

Nivel de educación del **PADRE** (marcar con una x según corresponda), incluya el número de años del último nivel alcanzado:

No sabe leer ni escribir___ Primaria incompleta___ Primaria completa___ Secundaria incompleta____ Secundaria completa___ Educación universitaria incompleta___ Educación universitaria completa____ Cuarto nivel___ Otro(Indique cual)_______

Número de años, último nivel educativo alcanzado:______

DATOS GENERALES DE LA NIÑA/O

a Fecha de Nacimiento de la niña/o: __ / __ / ____ (dd/mm/aaaa)

b Edad en años cumplidos de la niña/o: _____ Es él/ella hijo/a único/a: ☐ Si ☐ No

c Si **NO ES HIJA/O ÚNICA/O** ¿cuántas/os hermanas/os tiene? _______

d Orden de nacimiento de la niña/o ____

e ¿Todas las hermanas, incluida la/el niña/o tienen la misma madre y padre biológicos?
☐ Si ☐ No

f Nivel de educación actual de la niña/o: Educación inicial o preescolar ( ), educación general básica (indique nivel) ( ) ____, no va a la escuela ( )

Si él/ella va a la escuela, ¿cuántos días a la semana? ____

¿A qué hora entre y sale de la escuela regularmente? Hora entrada: __:__, Hora salida:__:__

g Sexo biológico de la niña/o (marcar con una x según corresponda):
Hombre ___ Mujer ___

h Auto-identificación étnica (marcar con una x según corresponda):
Afroecuatoriano/a___ Blanco/a____ Indígena___ Mestizo/a___ Montubio/a___
Mulato/a___ Negro/a___ Otro (Cuál)______________

i Ingresos económicos aproximados de los que dispone la familia para sus gastos, tomando en cuanta todos los aportes dentro y fuera del núcleo familiar: Menos de $530 ( ), entre $531 y $1060 ( ), entre $1061 y $1590 ( ), entre $1591 y $2120 ( ), más del$2120

j ¿Cuántas personas viven en la misma casa y comparten los gastos, incluida la niña/o?
____

4. ENCUESTA NIVEL SOCIOECONÓMICO DEL HOGAR

CÓDIGO NIÑA/O (a llenarse por la investigadora): _____________

Nombres y apellidos de la niña/o:____

NOTA: *este cuestionario será llenado por el padre, madre o cuidadora principal de la niña.*

Marque con una X en cada pregunta, lo que corresponda a su situación:

A CARACTERÍSTICAS DE LA VIVIENDA

a ¿Cuál es el tipo de vivienda donde vive?

Suite de lujo ☐ Cuarto(s) en casa de inquilinato ☐ Departamento en casa o edificio ☐

Casa/Villa ☐ Mediagua ☐ Rancho ☐ Choza/ Covacha/Otro ☐

b El material predominante de las paredes exteriores de la vivienda es de:

Hormigón ☐ Ladrillo o bloque ☐ Adobe/ Tapia ☐ Caña revestida o bahareque/ Madera ☐

Caña no revestida/ Otros materiales ☐

c El material predominante del piso de la vivienda es de:

Duela, parquet, tablón o piso flotante ☐ Cerámica, baldosa, vinil o marmetón ☐

Ladrillo o cemento ☐ Tabla sin tratar ☐ Tierra/ Caña/ Otros materiales ☐

d ¿Cuántos cuartos de baño con ducha de uso exclusivo tiene este hogar?

No tiene cuarto de baño exclusivo con ducha en el hogar ☐

Tiene 1 cuarto de baño exclusivo con ducha ☐

Tiene 2 cuartos de baño exclusivos con ducha ☐

Tiene 3 o más cuartos de baño exclusivos con ducha ☐

e El tipo de servicio higiénico con que cuenta este hogar es:

No tiene ☐ Letrina ☐ Con descarga directa al mar, río, lago o quebrada ☐

Conectado a pozo ciego ☐ Conectado a pozo séptico ☐ Conectado a red pública de alcantarillado ☐

B ACCESO A TECNOLOGÍA

a ¿Tiene este hogar servicio de internet?

Si ☐ No ☐

b ¿Tiene computadora de escritorio?

Si ☐ No ☐

c ¿Tiene computadora portátil?

Si ☐ No ☐

d ¿Cuántos celulares activados tienen en este hogar?

No tiene celular nadie en el hogar ☐ Tiene 1 celular ☐ Tiene 2 celulares ☐

Tiene 3 celulares ☐ Tiene 4 ó más celulares ☐

e ¿Tiene este hogar servicio de teléfono convencional?

Si ☐ No ☐

f ¿Tiene cocina con horno?

Si ☐ No ☐

g ¿Tiene refrigeradora?

Si ☐ No ☐

h ¿Tiene lavadora?

Si ☐ No ☐

i ¿Tiene equipo de sonido?

Si ☐ No ☐

j ¿Cuántos TV a color tienen en este hogar?

No tiene TV a color en el hogar ☐ Tiene 1 TV a color ☐ Tiene 2 TV a color ☐
Tiene 3 ó más TV a color ☐

k ¿Cuántos vehículos de uso exclusivo tiene este hogar?

No tiene vehículo exclusivo para el hogar ☐ Tiene 1 vehículo exclusivo ☐
Tiene 2 vehículo exclusivo ☐ Tiene 2 vehículo exclusivo ☐
Tiene 3 ó más vehículos exclusivos ☐

l ¿Alguien en el hogar compra vestimenta en centros comerciales?

Si ☐ No ☐

m ¿En el hogar alguien ha usado internet en los últimos 6 meses?

Si ☐ No ☐

n ¿En el hogar alguien utiliza correo electrónico que no es del trabajo?

Si ☐ No ☐

o ¿En el hogar alguien está registrado en una red social?

Si ☐ No ☐

p Exceptuando los libros de texto o manuales de estudio y lecturas de trabajo. ¿Alguien del hogar ha leído algún libro completo en los últimos 3 meses?

Si ☐ No ☐

q ¿Cuál es el nivel de instrucción del jefe del hogar?

Sin estudios ☐ Primaria incompleta ☐ Primaria completa ☐ Secundaria incompleta ☐
Secundaria completa ☐ Hasta 3 años de educación superior ☐
4 ó más años de educación superior (sin post grado) ☐ Post grado ☐

r ¿Alguien en el hogar está afiliado o cubierto por el seguro del IESS (general, voluntario o campesino) y/o seguro del ISSFA o ISSPOL?

Si ☐ No ☐

s ¿Alguien en el hogar tiene seguro de salud privada con hospitalización, seguro de salud privada sin hospitalización, seguro internacional, seguros municipales y de Consejos Provinciales y/o seguro de vida?

Si ☐ No ☐

t **¿Cuál es la ocupación del jefe o jefa del hogar? Escriba y luego seleccione el más adecuado**

____

Personal directivo de la Administración Pública y de empresas ☐
Profesionales científicos e intelectuales ☐ Técnicos y profesionales de nivel medio ☐
Empleados de oficina ☐ Trabajador de los servicios y comerciantes ☐
Trabajadores calificados agropecuarios y pesqueros ☐ Oficiales operarios y artesanos ☐
Operadores de instalaciones y máquinas ☐ Trabajadores no calificados ☐
Fuerzas Armadas ☐ Desocupados ☐ Inactivos ☐

u **Tiene agua potable**

Si ☐ No ☐

5. USO DE MEDICAMENTOS Y ANTECEDENTES MÉDICOS DE LA NIÑA/O

CÓDIGO NIÑA/O: ____________

Nombres y apellidos de la niña/o:____

> ***NOTA:*** *este cuestionario será administrado por personal capacitado del proyecto vía telefónica o en persona, en la escuela o vivienda dependiendo de la decisión de la representante legal.*

a Durante el **ÚLTIMO MES**, ha tomado alguna de las siguientes medicinas?
Medicina para tratar bacterias ( ); medicina para tratar virus ( ); medicina para tratar parásitos intestinales ( ); otra medicina ( ); no ha tomado ninguna medicina ( )
Nota: si responde que no ha tomado ninguna medicina, pase a la pregunta f

b Si la **RESPUESTA FUE SI** a alguno de las medicinas antes indicadas incluya la siguiente información:
Antibiótico (medicina para tratar bacterias): Escriba el nombre _______________________¿Cuántos días tomó? ________¿Cada qué tiempo? ___________¿Cuánto tomó (cucharadas grandes o pequeñas o tabletas o cualquier otra presentación):_________________ ¿Por qué motivo tomó? __________________________
Antiviral (medicina para tratar virus): Escriba el nombre _______________________¿Cuántos días tomó? ________¿Cada qué tiempo? ___________¿Cuánto tomó (cucharadas grandes o pequeñas o tabletas o cualquier otra presentación):_________________ ¿Por qué motivo tomó? __________________________
Antiparasitario (medicina para tratar parásitos): Escriba el nombre _______________________¿Cuántos días tomó? ________¿Cada qué tiempo? ___________¿Cuánto tomó (cucharadas grandes o pequeñas o tabletas o cualquier otra presentación):_________________ ¿Por qué motivo tomó?__________________________

Otra medicina: Escriba el nombre _______________________¿Cuántos días tomó? ________¿Cada qué tiempo? ___________¿Cuánto tomó (cucharadas grandes o pequeñas o tabletas o cualquier otra presentación):_________________ ¿Por qué motivo tomó?__________________________

c Debido a problemas de salud del niño o niña, acude de manera frecuente al médico o centro de salud:
Si ☐ No ☐ En caso de contestar que si:
¿Qué problema de salud tiene la niña/niño?____
¿Cada qué tiempo acude al médico o centro de salud?____

d Debido a problemas de salud del niño o niña, tiene que tomar alguna medicina de manera frecuente?
Si ☐ No ☐ En caso de contestar que sí:
¿Por qué motivo toma esa medicina?
____
¿Qué medicina toma? ¿Cada qué tiempo (horas/días/meses)?
____

e En el último mes ¿su niño o niña ha tomado alguna medicina natural por algún motivo?: Si ( ), No ( )
En caso de haber respondido sí:
¿Por qué motivo tomó esa medicina natural?________________________Escriba el nombre de la medicina natural _______________________¿Cuántos días tomó? ________¿Cada qué tiempo? ___________ ¿Cuánto tomó (cucharadas grandes o pequeñas o tabletas o cualquier otra presentación):_________________

f Durante el **ÚLTIMO AÑO** ¿su niño o niña ha tomado alguna de las siguientes medicinas? Medicina para tratar bacterias ( ); medicina para tratar virus ( ); medicina para tratar parásitos intestinales ( ); otra medicina ( ); no ha tomado ninguna medicina ( )
Nota: si responde que no ha tomado ninguna medicina, termine este cuestionario

g Si la **RESPUESTA FUE SI** a alguna de las opciones de la pregunta anterior (f), indique lo siguiente: cuántos tratamientos en el año_____________; el motivo del/los tratamiento/s__________________________; indique si el tratamiento fue recomendado por un médico/enfermero ( ) u otra persona ( ), indique quien_________________________

h En comparación con niños o niñas de la misma edad, diría que su niña o niño tiene **dificultad para ver**? No tiene ninguna ( ); tiene cierta dificultad ( ); tiene mucha dificultad ( ), le resulta imposible( )

i En comparación con niños o niñas de la misma edad, diría que su niña o niño tiene **dificultad para oír**? No tiene ninguna ( ); tiene cierta dificultad ( ); tiene mucha dificultad ( ), le resulta imposible ( )

j En comparación con niños o niñas de la misma edad, diría que su niña o niño tiene **dificultad para caminar**? No tiene ninguna ( ); tiene cierta dificultad ( ); tiene mucha dificultad ( ), le resulta imposible ( )

k En comparación con niños o niñas de la misma edad, diría que su niña o niño tiene **dificultad para comer, vestirse, ir al baño solo/a**? No tiene ninguna ( ); tiene cierta dificultad ( ); tiene mucha dificultad ( ), le resulta imposible ( )

l En comparación con niños o niñas de la misma edad, diría que su niña o niño cuando habla tiene **dificultad para que le entiendan** personas fuera del hogar? No tiene ninguna ( ); tiene cierta dificultad ( ); tiene mucha dificultad ( ), le resulta imposible ( )

m En comparación con niños o niñas de la misma edad, diría que su niña o niño tiene **dificultad para aprender o recordar cosas**? No tiene ninguna ( ); tiene cierta dificultad ( ); tiene mucha dificultad ( ), le resulta imposible ( )

n En comparación con niños o niñas de la misma edad, diría que su niña o niño tiene **dificultad para concentrarse en cosas que le gustan**? No tiene ninguna ( ); tiene cierta dificultad ( ); tiene mucha dificultad ( ), le resulta imposible ( )

o En comparación con niños o niñas de la misma edad, diría que su niña o niño tiene **dificultad para controlar su comportamiento**? No tiene ninguna ( ); tiene cierta dificultad ( ); tiene mucha dificultad ( ), le resulta imposible ( )

6. DIARIO DE ALIMENTOS DE LA NIÑA/O

CÓDIGO NIÑA/O: _____________

Nombres y apellidos de la niña/o:____

> ***NOTA:*** *este cuestionario será administrado por personal capacitado del proyecto vía telefónica o en persona, en la escuela o vivienda dependiendo de la decisión de la representante legal.*

Durante un día completo, dígame que alimentos consume su niño o niña. Trate de ser lo más especifico posible. Para evaluar cantidad de alimentos por usar el Manual Fotográfico de Porciones para Cuantificación Alimentaria – Ecuador 1era Edición.

Hora:	Tiempo de comida	Preparación	Listado de alimentos (tipo)	Cantidad de alimento
	Desayuno			
	Entre comida			
	Almuerzo			
	Entre comida			
	Cena			

7. ENCUESTA HABILIDADES SOCIALES (AQ-10 versión infantil)

CÓDIGO NIÑA/O (a llenar por la investigadora): _____________

Nombres y apellidos de la niña/o:____

> ***NOTA:*** *este cuestionario será llenado por el padre, madre o cuidadora principal de la/el niña/o.*

A continuación se solicita que marque **CON UNA X SOLAMENTE UNA DE LAS OPCIONES**, la que crea más adecuada para dar respuesta a cada una de las preguntas. Recuerde **NO EXISTEN RESPUESTAS EQUIVOCADAS.**

		Totalmente de acuerdo	Parcialmente de acuerdo	Parcialmente en desacuerdo	Totalmente en desacuerdo
1	La niña/niño con frecuencia escucha sonidos que otros no escuchan.				
2	La niña/niño tiende a ver todo, en lugar de concentrarse en los pequeños detalles.				
3	Estando en grupo, la niña/niño fácilmente puede seguir las conversaciones de personas diferentes.				
4	A la niña/niño le resulta fácil pasar de una actividad a otra.				
5	La niña/niño no sabe cómo mantener una conversación con sus compañeras/compañeros.				
6	La niña/niño es buena/bueno socializando y conversando.				
7	A la niña/niño le resulta difícil entender las intenciones o sentimientos de los personajes cuando se le lee un cuento o se le cuenta una historia.				
8	Cuando la niña/niño estuvo en preescolar, disfrutó jugar con otras/otros niñas/niños interpretando personajes o asumiendo roles.				
9	A la niña/niño le resulta fácil entender lo que alguien está pensando o sintiendo con sólo mirar su cara.				
10	A la niña/niño le resulta difícil hacer nuevos amigos.				

8. ENCUESTA EVALUACIÓN DEL SUEÑO DE LA NIÑA/O (Escala Bruni)

CÓDIGO NIÑA/O (a llenar por la investigadora): _____________

Nombres y apellidos de la niña/o:____

NOTA: *este cuestionario será llenado por el padre, madre o cuidadora principal de la niña.*

A continuación se solicita que marque **CON UNA X SOLAMENTE UNA DE LAS OPCIONES**, la que crea más adecuada para dar respuesta a cada una de las preguntas. Recuerde **NO EXISTEN RESPUESTAS EQUIVOCADAS.**

**1 ¿Cuántas horas duerme la mayoría de las noches?**				
9–11 horas ( )	8–9 horas ( )	7-8 horas ( )	5-7 horas ( )	Menos de 5 horas ( )
**2 ¿Cuánto tarda en dormirse?**				
Menos de 15 minutos ( )	15-30 minutos ( )	30-45 minutos ( )	45-60 minutos ( )	Más de 60 minutos ( )

		Nunca	Ocasionalmente (1-2 veces al mes)	Algunas veces (1-2 veces por semana)	A menudo (3-5 veces por semana)	Siempre (diariamente)
3	Se va a la cama de mal humor.					
4	Tiene dificultad para quedarse dormido por la noche.					
5	Parece ansioso o miedoso mientras se queda dormido.					
6	Sacude o agita partes del cuerpo mientras se queda dormido.					
7	Realiza movimientos repetitivos, tales como mover la cabeza o rodarse por la cama mientras se queda dormido.					
8	Tiene imágenes de sus sueños que parecen reales , es decir “tiene sueños vívidos”, mientras se queda dormido.					
9	Suda excesivamente al dormirse.					
10	Se despierta más de dos veces por la noche.					

11	Después de despertarse por la noche, tiene dificultades para dormirse nuevamente.					
12	Tiene sacudidas en las piernas o cambia a menudo de posición o patea las cobijas de la cama mientras duerme.					
13	Tiene dificultades para respirar durante la noche.					
14	Se queda sin aliento o no puede respirar mientras duerme.					
15	Ronca.					
16	Suda excesivamente durante la noche.					
17	Usted ha observado que camina dormido.					
18	Usted ha observado que habla dormido.					
19	Rechina los dientes dormido.					
20	Se despierta gritando o confundido o fuera de sí durante la noche, aunque no lo recuerda al día siguiente.					
21	Tiene pesadillas que no recuerda al día siguiente.					
22	Es muy difícil despertarlo por la mañana.					
23	Se despierta cansado por la mañana.					
24	Parece que no puede moverse al despertarse por la mañana.					
25	Está adormilado (somnoliento) durante el día.					
26	Se duerme de repente en situaciones o momentos inapropiados.					

9. ENCUESTA EVALUACIÓN ACTIVIDAD FÍSICA DE LA NIÑA/O (IPA-Q)

CÓDIGO NIÑA/O (a llenar por la investigadora): _____________

Nombres y apellidos de la niña/o:____

NOTA: *este cuestionario será llenado por el padre, madre o cuidadora principal de la niña.*

A continuación se solicita que marque **ESCRIBA UNA RESPUESTA O MARQUE CON UNA X SEGÚN CREA CONVENIENTE**. Recuerde **NO EXISTEN RESPUESTAS EQUIVOCADAS.**

**1. Durante los últimos 7 días, ¿en cuántos realizó actividades físicas intensas tales como levantar pesos pesados, cavar, ejercicios hacer aeróbicos o andar rápido en bicicleta?**	
Días por semana (indique el número)	
Ninguna actividad física intensa (pase a la pregunta 3)	
**2. Habitualmente, ¿cuánto tiempo en total dedicó a una actividad física intensa en uno de esos días?**	
Indique cuántas horas por día	
Indique cuántos minutos por día	
No sabe/no está seguro	
**3. Durante los últimos 7 días, ¿en cuántos días hizo actividades físicas moderadas tales como transportar pesos livianos, o andar en bicicleta a velocidad regular? No incluya caminar**	
Días por semana (indicar el número)	
Ninguna actividad física moderada (pase a la pregunta 5)	
**4. Habitualmente, ¿cuánto tiempo en total dedicó a una actividad física moderada en uno de esos días?**	
Indique cuántas horas por día	
Indique cuántos minutos por día	
No sabe/no está seguro	
**5. Durante los últimos 7 días, ¿en cuántos días caminó por lo menos 10 minutos seguidos?**	
Días por semana (indicar el número)	
Ninguna caminata (pase a la pregunta 7)	
**6. Habitualmente, ¿cuánto tiempo en total dedicó a caminar en uno de esos días?**	
Indique cuántas horas por día	
Indique cuántos minutos por día	
No sabe/no está seguro	
**7. Durante los últimos 7 días, ¿cuánto tiempo pasó sentado durante un día hábil?**	
Indique cuántas horas por día	
Indique cuántos minutos por día	
No sabe/no está seguro	
