# Supplementary material for: Microbiota, parasitic infections and their relationship with nutritional status and neurocognitive functioning in children from Ecuador—Proyecto Guagua: research protocol for a cross-sectional study
Source: Front Public Health. 2025 Jan 29;13:1505780. doi: 10.3389/fpubh.2025.1505780 (PMC11815663; doi:10.3389/fpubh.2025.1505780)
Supplement: Supplementary file 2 [file Data_Sheet_2.pdf]

10. CUESTIONARIO PARA LA ESCUELA (FICHA DE OBSERVACIÓN)

Nota: Este cuestionario debe ser llenado por escuela, después de observar las instalaciones y hablar con la persona encargada y autorizada para proporcionar dicha información.

1. Nombre de la escuela:____

2. Dirección de la escuela: Calle principal:____

Calle secundaria:_____________________________ Localidad:________________________

Cantón:______________________ Provincia________________________

3. Número de alumnos:_______________

4. Niveles de educación que ofrece:______________

5. Número de profesores:__________________

6. Número de personal administrativo a parte de los profesores:___________________

7. ¿Cuál es la principal fuente de agua para consumir o beber en la escuela *(marcar solo la que se usa con más frecuencia)* Agua entubada potable ( ), agua entubada no potable ( ), pozo o manantial protegido y monitoreado para evitar contaminación ( ), agua de lluvia( ), pozo o manantial no protegido o no monitoreado( ), agua envasada ( ), carro o camión cisterna ( ), agua de superficie (lago, río, arroyo) ( ), sin fuente de agua ( )

8. ¿Dispone la escuela actualmente de agua de consumo procedente de la fuente principal (hace referencia a la pregunta anterior)? Si ( ), No ( )

9. ¿De qué tipo de inodoros o letrinas disponen los alumnos y alumnas? *(marcar solo la que se usa con más frecuencia)* Inodoros de arrastre hidráulico (baño normal)( ), letrinas de pozo excavado con losa ( ), letrinas de compostaje ( ), letrinas de pozo excavado sin losa ( ), otro tipo de letrina ( ) indique cual ____, no hay inodoros, ni letrinas ( )

10. ¿Cuántos de los inodoros o letrinas para los alumnos o alumnas son utilizables actualmente (disponibles, en funcionamiento o privados) (indique el número de tazas o asientos o compartimientos) ________

11. ¿Los inodoros o las letrinas están separados por sexo? Si ( ), No ( )

12. ¿Dispone la escuela de instalaciones para el lavado de manos? Si ( ), No ( )

13. ¿Disponen actualmente de agua y jabón las instalaciones par el lavado de manos? Si, agua y jabón ( ), solo agua ( ), solo jabón ( ), ni agua ni jabón ( )
