# Supplementary material for: Microbiota, parasitic infections and their relationship with nutritional status and neurocognitive functioning in children from Ecuador—Proyecto Guagua: research protocol for a cross-sectional study
Source: Front Public Health. 2025 Jan 29;13:1505780. doi: 10.3389/fpubh.2025.1505780 (PMC11815663; doi:10.3389/fpubh.2025.1505780)
Supplement: Supplementary file 3 [file Supplementary_file_3.pdf]

Supplementary information 3

1. Processing of stool samples for the analysis of the gut microbiota

Collected stool samples will be processed to isolate total genomic DNA (gDNA). For bacterial cell lysis, approximately 250 mg of fecal sample will be transferred to a ZR bashingbead™ Lysis tube containing 0.1- and 0.5-mm silica beads. Next, 750 µL of BashingBead™ buffer (Zymo Research, Catalogue No. D6001-3) will be added, followed by homogenization in a vortex mixer for 40 min as per the manufacturer's recommendations. The gDNA will be purified using the Quick-DNA Fecal/Soil Microbe Miniprep Kit (Zymo Research, Catalogue No. D6010) according to the manufacturer's procedure. The quality and quantity of the isolated gDNA will be assessed using a Nanodrop spectrophotometer and a Qubit 4 Fluorometer (Thermo Fisher Scientific).

Sequencing libraries will be constructed by amplifying the V4 hypervariable region of the 16S rRNA bacterial gene. This process involves two PCR reactions using the OneTaq Hot Start DNA Polymerase (New England Biolabs Inc., Catalogue No. M0481). The first PCR reaction (PCR-1) amplifies the V4 region and attaches Illumina adapter overhang sequences using the 16S_515F forward primer and the 16S_806R reverse primer. PCR-1 mix will consist of 1 µL of 1 ng to 1 µg gDNA, 5 µL of OneTaq Hot Start 2X Master Mix in Standard Buffer (New England BioLabs Inc., Catalogue No. M0484), 0.4 µL of each 10 µM forward and reverse primer, and 3.2 µL of PCR-grade water, achieving a total reaction volume of 10 µL per reaction. The PCR will be performed using a SimpliAmp Thermal Cycler (Applied Biosystems™) with the following parameters: 1 cycle of 5-min initial denaturation at 94°C, 10 cycles of 15-s denaturation at 94°C, 30-s annealing at 50°C, 30-s extension at 68°C, and 1 cycle of 10-min final extension at 68°C. The expected amplicon size from PCR-1 will be 390 bp.

The second PCR reaction (PCR-2) will add the i5 and i7 index adapters to PCR-1 products. PCR-2 will be prepared by mixing 1 µL PCR-1 product, 5 µL OneTaq Hot Start 2X Master Mix in Standard Buffer (New England BioLabs Inc., Catalog No. M0484), 0.5 µL of each 10 µM i5 and i7 index primers, and 3 µL PCR-grade water, resulting in a total reaction volume of 10 µL per reaction. PCR-2 will be performed using SimpliAmp Thermal cycler (Applied Biosystems™) with the following parameters: 1 cycle of 5-min initial denaturation at 95°C, 30 cycles of 15-s denaturation at 95°C, 30-s annealing at 55°C and 30-s extension at 68°C, and 1 cycle of 5-min final extension at 68°C.

Following PCR-2, sequencing libraries will be pooled and cleaned using the AMPure XP Beads protocol (Agencourt Bioscience Corporation, Beverly, MA, USA) at a 1X ratio. The quantity of the final sequencing libraries will be determined using a Qubit 4 Fluorometer, while amplicon size verification will be performed by gel electrophoresis and the Agilent 2100 Bioanalyzer System (Agilent Technologies, Palo Alto, CA). Finally, these libraries will be sequenced in a 2 × 300 bp paired-end run using the Illumina MiSeq Platform. The sequencing procedure will be performed in triplicate.
