# Supplementary material for: Microbiota, parasitic infections and their relationship with nutritional status and neurocognitive functioning in children from Ecuador—Proyecto Guagua: research protocol for a cross-sectional study
Source: Front Public Health. 2025 Jan 29;13:1505780. doi: 10.3389/fpubh.2025.1505780 (PMC11815663; doi:10.3389/fpubh.2025.1505780)
Supplement: Supplementary file 4 [file Table_1.pdf]

Supplementary Table 1. Distribution of participating schools and social organizations.
Symbols and abbreviations: SO, social organization, S, school.

**School/organization**	**Localization**	**Public/private**
SO1	Urban, Riobamba, Chimborazo, Highlands	Private
SO2	Urban, Riobamba, Chimborazo, Highlands	Private
S1	Urban, Pallatanga, Chimborazo, Highlands	Public
S2	Urban, Puyo, Pastaza, Amazon	Public
S3	Rural, Colta, Chimborazo, Highlands	Public
SO3	Urban, Penipe, Chimborazo, Highlands	Public
S4	Rural, Penipe, Chimborazo, Highlands	Public
S5	Rural, Penipe, Chimborazo, Highlands	Public
S6	Rural, Penipe, Chimborazo, Highlands	Public
S7	Rural, Penipe, Chimborazo, Highlands	Public
S8	Urban, Playas, Guayas, Coast	Public
SO4	Rural, Guayaquil, Guayass, Coast	Public
SO5	Urban, Guayaquil, Guayas, Coast	Private
S9	Urban, San Cristóbal, Galapagos	Public
